# Differential NPY-Y1 Receptor Density in the Motor Cortex of ALS Patients and Familial Model of ALS

**DOI:** 10.3390/brainsci11080969

**Published:** 2021-07-23

**Authors:** Courtney M. Clark, Rosemary M. Clark, Joshua A. Hoyle, Jyoti A. Chuckowree, Catriona A. McLean, Tracey C. Dickson

**Affiliations:** 1 Menzies Institute for Medical Research, College of Health and Medicine, University of Tasmania, 17 Liverpool Street, Hobart, TAS 7000, Australia; Courtney.Clark@utas.edu.au (C.M.C.); Rosemary.Clark@utas.edu.au (R.M.C.); Joshua.Hoyle@utas.edu.au (J.A.H.); Jyoti.Chuckowree@utas.edu.au (J.A.C.); 2 Anatomical Pathology, Alfred Health, The Alfred Hospital, Melbourne, VIC 3181, Australia; C.McLean@alfred.org.au

**Keywords:** motor cortex, upper motor neurons, amyotrophic lateral sclerosis, neuropeptide Y

## Abstract

Destabilization of faciliatory and inhibitory circuits is an important feature of corticomotor pathology in amyotrophic lateral sclerosis (ALS). While GABAergic inputs to upper motor neurons are reduced in models of the disease, less understood is the involvement of peptidergic inputs to upper motor neurons in ALS. The neuropeptide Y (NPY) system has been shown to confer neuroprotection against numerous pathogenic mechanisms implicated in ALS. However, little is known about how the NPY system functions in the motor system. Herein, we investigate post-synaptic NPY signaling on upper motor neurons in the rodent and human motor cortex, and on cortical neuron populations in vitro. Using immunohistochemistry, we show the increased density of NPY-Y1 receptors on the soma of SMI32-positive upper motor neurons in post-mortem ALS cases and SOD1^G93A^ excitatory cortical neurons in vitro. Analysis of receptor density on *Thy1-YFP-H*-positive upper motor neurons in wild-type and SOD1^G93A^ mouse tissue revealed that the distribution of NPY-Y1 receptors was changed on the apical processes at early-symptomatic and late-symptomatic disease stages. Together, our data demonstrate the differential density of NPY-Y1 receptors on upper motor neurons in a familial model of ALS and in ALS cases, indicating a novel pathway that may be targeted to modulate upper motor neuron activity.

## 1. Introduction

The motor cortex is a region of the frontal lobe responsible for learning and planning of complex motor sequences and relay of behavioral and environmental cues to modify movements elicited by downstream motor pathways. To maintain long-term stability and function of motor networks, a physiological balance between facilitatory and inhibitory circuitry is established, known as the excitation/inhibition (E/I) ratio. The E/I ratio is shaped by a heterogeneous network of different cell types, receptors and signaling mechanisms [1], and changes to any one of these components can trigger significant effects on the overall function and activity of the corticomotor system.

In the fatal motor neuron disease, amyotrophic lateral sclerosis (ALS), the destabilization of faciliatory and inhibitory signaling represents an important aspect of corticomotor pathology. Transcranial magnetic stimulation studies demonstrate that changes in the E/I state of brain networks contribute to cortical hyperexcitability in the motor cortex of patients, preceding lower motor neuron loss, and coinciding with the emergence of motor symptoms [2,3,4]. There is increased interest in determining the exact mechanisms of cortical hyperexcitability in ALS, as recent studies indicate that it may be a critical determinant of disease progression and therefore a therapeutic target capable of delaying or halting the disease [5,6]. However, the exact etiology behind disrupted cortical network excitability is unclear. Intrinsic factors such as modified ionotropic receptors, synapses, and calcium signaling have been implicated in the intrinsic hyperexcitability of upper motor neurons demonstrated in models of ALS [7,8,9,10,11,12,13,14]. However, there is also compelling evidence that a loss of effective inhibition is a major cause of cortical network hyperexcitability in ALS [15,16,17,18,19].

Cortical inhibition is facilitated by a heterogenous population of interneurons [20], which are essential for regulating activity of excitatory and inhibitory networks within the primary motor cortex [21,22,23]. In animal models of ALS, evidence suggests that there is a loss of inhibitory influence from the interneurons that directly synapse onto upper motor neurons and regulate their excitability [19,24]. Studies that have reversed this loss of inhibitory function by increasing the activity of layer 5 interneurons have not only prevented upper motor neuron hyperexcitability, but also significantly delayed the onset of motor symptoms [24]. Indeed, clinical imaging studies indicate that reduced inhibitory activity in the ALS motor cortex coincides with cortical hyperexcitability and strongly associates with disease severity in patients [3,5]. In view of the critical role of the inhibitory system in the pathogenesis of ALS (see review [25]), it is surprising that little is known about the role of other key neuromodulators that may influence excitability in the disease.

Neuropeptide Y (NPY) signaling has been extensively associated with modulation of pre- and post-synaptic excitatory potentials across multiple brain regions, and has been shown to be neuroprotective by improving motor deficits and survival in models of neurodegenerative disease (see review [26]). Importantly, multiple studies have implicated the NPY system in the ALS disease pathogenesis. Investigators have shown that changes to NPY interneurons occur within the motor cortex of the *SOD1^G93A^* familial ALS mouse model throughout the disease course [27]. NPY cells were found to significantly decrease in number during early symptom onset and increase in number at end stage of disease suggesting that NPY is involved with, or contributes to, disease processes that evolve within the ALS motor cortex [27]. Human studies have also revealed decreased NPY-positive fibers in the motor cortex of ALS cases with a high degree of Betz cell (upper motor neuron) depletion. Moreover, increased NPY levels detected in the blood of ALS cases were associated with shorter disease duration [28], indicating a role for NPY in underlying patterns of neurodegeneration. Importantly, NPY can exert a significant neuroprotective effect to reduce elevated levels of neuronal excitability in human brain networks [29]. The increased synthesis and release of NPY are widely reported in animal models and patients with epilepsy [30,31,32], suggesting that NPY signaling could also modulate key aspects of the cortical E/I imbalance during critical stages of ALS disease pathogenesis.

Based on protective effects of NPY in neuronal networks, NPY signaling is increasingly considered a novel therapeutic target for neurodegenerative and neurological disorders (see review [33]). However, there is relatively little known about the NPY system in the ALS motor cortex. To determine the clinical value of NPY for ALS, there is a need to better understand if NPY signaling is affected in this critical disease-associated area. Predominately released by GABAergic interneurons, NPY signals through a multi-ligand/receptor system, evoking a complex range of biological actions through binding to, and activating, various receptor subtypes (Y1, Y2, Y4, Y5, and Y6). In the cortex, NPY interneurons and Y receptors are present throughout all cortical layers [34,35,36]. However, the primary site of NPY action on layer 5 pyramidal neurons is suggested to occur through the post-synaptic NPY-Y1 receptors [37,38].

As such, the primary goal of the current study was to determine whether there are changes to the expression patterns of NPY-Y1 receptors on upper motor neurons within the diseased motor cortex, and if so, at what stage in disease they become altered. To this end, we investigated NPY-Y1 receptors on upper motor neurons in the motor cortex of post-mortem ALS cases and in the *SOD1^G93A^* familial mouse model of ALS, and on *SOD1^G93A^* excitatory neocortical neurons in vitro.

## 2. Materials and Methods

### 2.1. Animals

All experiments requiring the use of animals were reviewed and approved by the Animal Ethics Committee of the University of Tasmania and conducted in accordance with the Australian Code of Practice for the Care and Use of Animals for Scientific Purposes, 2013.

Animals used in this study were housed in ventilated cages at 20 °C, on a 12 h light/dark cycle with food and water ad libitum. All mouse strains utilized in this study were backcrossed and maintained on a *C57BL/6* background. Transgenic male *SOD1^G93A^* mice carrying a high copy number of the human *SOD1^G93A^* mutation (B6.Cg-Tg(*SOD1^G93A^*)1.Gur.J/ Stock No: 004435; Jackson Laboratory, Bar Harbor, ME, USA) (accessed on 28 February 2019) [39] were mated with female *Thy1-YFP-H* mice (B6.Cg-Tg(*Thy1-YFP*)HJrs/J/ Stock No: 003782 (accessed on 27 November 2018); Jackson Laboratory, Bar Harbor, ME, USA) to generate *Thy1-YFP-H::SOD1^G93A^* double transgenic mice and *Thy1-YFP-H* single transgenic non-diseased controls. *Thy1-YFP-H* mice express yellow fluorescent protein (YFP) that is restricted to layer 5 pyramidal neurons of the cerebral cortex [40,41]. Only male *Thy1-YFP-H* and *Thy1-YFP-H::SOD1^G93A^* mice were selected for tissue experiments in the present study. Assessment of *SOD1^G93A^* gene copy number was performed externally by Transnetyx (Cordova, TN, USA).

For in vitro investigations, male SOD1^G93A^ mice were time mated with female *Thy1*-*YFP^+/+^* mice (B6.Cg-Tag(*Thy1-YFP*)16Jrs/J/ Stock No: 003709 (accessed on 28 February 19); Jackson Laboratory, Bar Harbor, ME, USA) on expressing cytosolic yellow fluorescent protein under the pyramidal neuron specific *Thy-1* promoter to generate double transgenics [40]. Littermates positive for *Thy1-YFP* but negative for *SOD1^G93A^* were utilized as experimental controls. Genotyping for the *SOD1^G93A^* gene for in vitro experiments was performed using quantitative polymerase chain reactions (qPCR). DNA was extracted from tails of individual embryos at E15.5 using an Extract-N-Amp tissue PCR tissue kit (Sigma Aldrich, Sydney, Australia) as per manufacturer’s instructions. Primers against apolipoprotein B (*ApoB*) gene were used as an internal DNA control. For qPCR, 50–100 ng of extracted DNA was added to the qPCR solution containing 500 nM *ApoB* forward and reverse primer mix (*ApoB* forward primer, 5′- CAC GTG GGC TCC AGCAT-3′; *ApoB* reverse primer, 5′- TCA CCA GTC ATT TCT GCC TTT G-3′) (IDT Technologies, San Diego, CA, USA), 150 nM *SOD1^G93A^* forward and reverse primer mix (*SOD1^G93A^* forward primer, 5′-GGG AAG CTG TTG TCC CAA G-3; *SOD1^G93A^* reverse primer, 5′-CAA GGG GAG-GTA AAA GAG AGC-3′) (IDT Technologies, San Diego, CA, USA), 0.15 µM Tmol ApoB (Hex TaqMan probe, IDT Technologies, San Diego, CA, USA), 0.15 µM Tmol SOD1 (6-FAM TaqMan probe, IDT Technologies, San Diego, CA, USA), 2xSensiFast SYBR no-ROX kit (Bioline, London, UK), and DNAase free water. qPCR amplification was implemented using the Rotor-Gene Q (Qiagen, Hilden, Germany).

### 2.2. Human Tissue Preparation

All procedures performed in studies involving human participants were in accordance with the ethical standards of the Tasmanian Health and Medical Research Ethics Committee (H0016154) of the University of Tasmania and fulfils the National Health and Medical Research Council (NHMRC) of Australia’s issued statement on human experimentation and is in accordance with the 1964 Helsinki declaration and its later amendments or comparable ethical standards. Human brain tissue was obtained via a tissue request to the Victorian and Sydney Brain Banks and fulfilled the following inclusion criteria: (1) primary clinical presentation of ALS and (2) availability of formalin-fixed primary motor cortex. Cases were anonymized, but information was provided regarding sex, age at death, and post-mortem interval summarized in (Table 1). Human brain sections were cut from formalin-fixed blocks of primary motor cortex, using cryosectioning techniques, as described below (Section 2.3).

### 2.3. Mouse Tissue Preparation

Cohorts of *YFP-H::SOD1^G93A^* (*n* = 6 per age group) and *YFP-H* mice (*n* = 6 per age group) were transcardially perfused with 4% paraformaldehyde solution (pH 7.4, Sigma Aldrich, Sydney, Australia) at time points reflective of early-symptomatic (8 weeks), and late-symptomatic (20 weeks) periods of the SOD1^G93A^ disease course. Perfused brains were dissected out and post-fixed in 4% paraformaldehyde overnight prior to storage at 4 °C in 0.01 M phosphate buffered saline (PBS) containing 0.1% *w/v* sodium azide (Sigma Aldrich, Sydney, Australia). In preparation for cryosectioning, brains were equilibrated in a series of increasing sucrose concentrations (4%, 16%, 30%) (Sigma Aldrich, Sydney, Australia) in 0.01 M PBS, for 24 h per concentration. Coronal sections were cut with a cryostat (Leica Biosystems, Melbourne, Australia) at a thickness of 30 μm. Sections were collected in sequential order and placed free-floating into 0.01 M PBS with 0.1% *w/v* sodium azide (Sigma Aldrich, Sydney, Australia) and stored at 4 °C.

### 2.4. Primary Cortical Neuronal Culture

At day 15.5 of embryo gestation, pregnant female mice were sacrificed using carbon dioxide (CO_2_) and embryos were removed. Cortices of single embryos were dissected under a light microscope and chemically and mechanically digested to form a single cell solution as described previously (*n* = minimum 8 embryos per group across five independent experiments) [42]. Dissociated cortical neurons were seeded onto 0.001% poly-L-lysine-coated 13 mm round coverslips within 24 well plates at a density of 37,500 cells/well. Neurons were grown in supplemented Neurobasal^TM^ medium (2% B27 supplement, 1% antimycotic-antibiotic, 0.25% Glutamax^TM^, 10% heat-inactivated fetal bovine serum and 0.5% 200X glutamic acid) overnight at 37 °C and 5% CO_2_. The following day the medium of neurons was replaced with “subsequent” Neurobasal^TM^ medium supplemented with 2% B27 supplement, 1% antimycotic-antibiotic, 0.25% Glutamax^TM^. All reagents were obtained from Thermo-Fisher Scientific (Melbourne, Australia). Neurons were maintained in subsequent medium until 14 days in vitro (DIV) at a constant temperature (37 °C) and CO_2_ (5%), with 100% of media replaced at 7 DIV. At 14 DIV neurons were fixed with 4% paraformaldehyde.

### 2.5. Immunocytochemistry and Immunohistochemistry

To assess NPY-Y1 receptor density on human upper motor neurons, motor cortex sections from de-identified ALS cases (*n* = 9) and controls (*n* = 6) were labelled according to standard protocols (adapted from [43]) optimized for formalin-fixed paraffin embedded neurological specimens. Antigens were unmasked in a 10 mM pH6 citric acid solution in the microwave at 640 W for 20 min and cooled in the citric solution for 30 min. Non-specific binding was blocked for up to 1 h in 3% normal goat serum in 0.3% TritonX-100 in phosphate-buffered saline (PBS) solution. Sections were incubated in blocking solution with primary antibodies overnight (Table 2). After washes, sections were incubated in fluorescent secondary antibodies (Table 2) for 2 h at room temperature. Autofluorescence was quenched with Vector TrueVIEW kit (3 min) and sections counterstained with NeuroTrace fluorescent Nissl-435/455 to visualize neuronal nuclei (1:50 Thermo-Fisher Scientific, Melbourne, Australia; N21479).

For assessment of NPY-Y1 receptor density in mouse tissue, a minimum of two free-floating sections per animal from bregma 0.38 to 0.02 mm containing primary motor cortex were selected for analysis. Sections were washed (3 × 10 min in 0.01 M PBS) and blocked with a protein block (Agilent Technologies, Melbourne, Australia) for 15 min. Primary antibody specific to the NPY-Y1 receptor was diluted in 0.01 M PBS containing 0.3% Triton-X-100 and incubated with sections overnight at 4 °C with agitation (Table 2 for dilution factor). After washing (3 × 10 min in 0.01 M PBS), sections were incubated with AlexaFluor conjugated secondary antibodies (Table 2) and a Nissl stain (1:50 dilution, Thermo-Fisher Scientific, Melbourne, Australia) for 90 min at room temperature with agitation.

For in vitro investigations, fixed neurons were permeabilized and blocked (0.3% TritonX in 0.01 M PBS, 5% goat serum) for 30 min at room temperature and incubated in primary antibodies diluted in 5% goat serum + 0.01 M PBS, overnight at 4 °C (Table 2). The following day, coverslips were washed (3 × 10 min) with 0.01 M PBS and incubated in Alexafluor conjugated secondary antibodies and nuclear stain DAPI (1:7500 in 0.01 M PBS) for 90 min at room temperature.

All tissue sections and coverslips were washed (3 × 10 min) in 0.01 M PBS and mounted onto glass slides using PermaFluor aqueous mounting medium (Thermo-Fisher Scientific, Melbourne, Australia).

### 2.6. Confocal Microscopy

Immunofluorescence was captured using a UltraVIEW VoX spinning disk confocal microscope, running Velocity software (v6.3.0, Perkin Elmer, Melbourne, Australia). In human motor cortex sections, nissl staining in conjunction with SMI32 labelling was used to demarcate cortical layers based on previously established methods [44,45]. In mouse cortical sections, prior to image acquisition, a 20×/0.345 air objective (Nikon, New York, NY, USA) was used to identify grey matter of the primary motor cortex based on the appearance of anatomical landmarks, as described previously [46]. In mouse cortical sections, nissl staining was used to visualize neuronal nuclei and in conjunction with YFP-H labelling was used to demarcate cortical layers for receptor analysis. For NPY-Y1 receptor analysis, a 60×/0.0767 water objective (Nikon, New York, NY, USA) was used to capture a minimum of 6 *z*-stack images (0.4 μm intervals) of YFP-H-positive neurons (mouse cortex) and SMI32-positive (human motor neurons) across layers 5, 2/3 and 1.

To assess NPY-Y1 receptor expression in vitro, a 60×/0.0767 water objective (Nikon, New York, NY, USA) was used to capture *z*-stack images (0.2 μm intervals) of YFP-positive cortical neurons. Per individual embryo a minimum of 30 cells across triplicate coverslips were captured. All image acquisition was completed blinded to genotype.

### 2.7. Image Analysis

**Figure 1:**
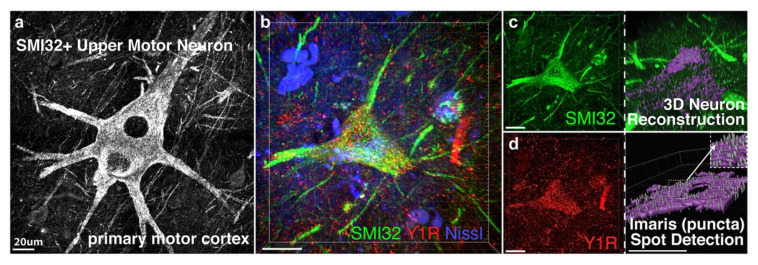
For NPY-Y1 receptor quantification upper motor neurons were visualized with the neurofilament protein SMI32 in human motor cortex (**a**), or with the fluorescent protein YFP-H in *YFP-H* rodent motor cortex, and reconstructed from z-stack images using Imaris software. Z-stack image showing an SMI32-positive upper motor neuron in post-mortem human tissue (green) labelled with the NPY-Y1 receptor (red) and the neuronal marker Nissl (blue) (**b**). NPY-Y1 receptor puncta/µm^3^ was determined on three-dimensional (3D) rendered neurons (purple) (**c**) using spot detection algorithms (**d**). Imaris object detection feature allowed for isolation of cell soma (**c**) or apical process compartments for puncta analysis per volume of object reconstructed. Insert in (**d**) shows synaptic puncta detected using Imaris. Scale bars = 20 µm.

NPY-Y1 receptor quantification was analyzed using Imaris ×64 (v9.2.0, Bitplane, Zürich, Switzerland) image analysis software (see Figure 1). Soma and neuronal processes were selected and 3D rendered from z-stacks using the “surface” function and receptors were selected with the “spots” function (0.5 µm size threshold). Quantification of receptors in contact with dendrites was performed using the “find spots close to surface” (0.5 µm distance threshold) extension (Matlab R2019a, MathWorks, Sydney, Australia). Volume measurements of selected dendrites and cell somas were taken to calculate NPY-Y1 receptor density per image (presented as puncta/µm^3^).

### 2.8. Statistical Analysis

Human tissue statistical analysis was performed using SPSS Statistics version 27 (IBM, New York, NY, USA). ALS cases and age-matched controls were compared for NPY-Y1 receptor density using analysis of variance (ANOVA). Additionally, two-way repeated-measures ANOVA was performed to assess differences in receptor expression between upper motor neuron compartments (soma, apical dendrite compartments). Fisher’s exact test was used to measure differences in dichotomous variables such as sex. All continuous data passed the Shapiro–Wilk normality test (Age, PMI, Y1 receptor density).

Rodent analyses were performed using Prism 9 (v 9.1.1, GraphPad, San Diego, CA, USA). *YFP* (control) and *YFP::SOD1^G93A^* cortical neurons were compared for NPY-Y1 receptor expression in vitro using ANOVA. In the rodent motor cortex, *YFP-H* (control) and *YFP-H::SOD1^G93A^* NPY-Y1 receptor expression was compared across age (8 weeks and 20 weeks) using two-way ANOVA, with Tukey’s post hoc tests performed to investigate differences between genotypes and age within individual upper motor neuron compartments (soma, apical dendrites). Additionally, three-way ANOVA analysis with Tukey’s post hoc test was performed to compare NPY-Y1 receptor puncta differences between upper motor neuron compartments, genotypes and time points. A *p* value of ≤ 0.05 was considered statistically significant for all analyses performed.

## 3. Results

### 3.1. NPY-Y1 Receptor Density Is Increased on SMI32-Positive Upper Motor Neurons in the Motor Cortex of ALS Cases

**Figure 2:**
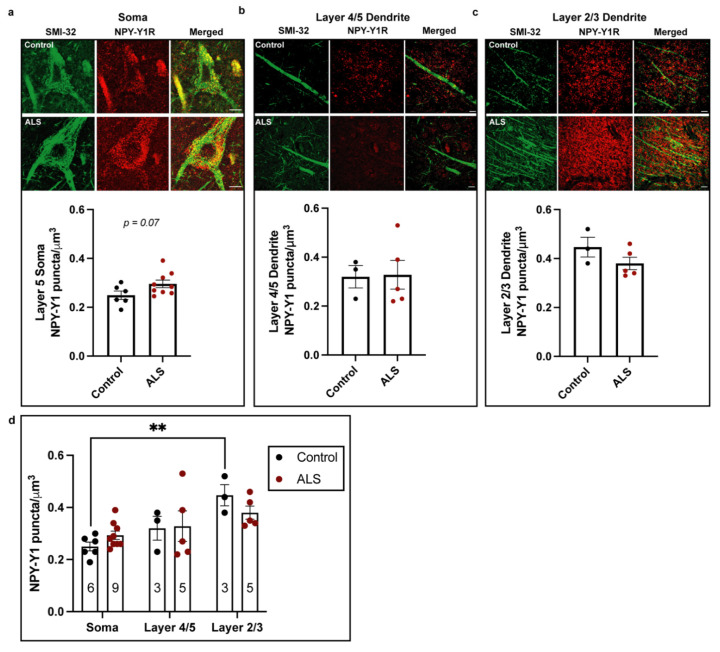
Immunohistochemical comparisons of NPY-Y1 receptors localized to the cell soma (**a**) and apical dendrites localized in cortical layer 4/5 (**b**) and 2/3 (**c**) of SMI32-positive upper motor neurons in the post-mortem motor cortex of ALS cases (*n* = 9) and controls (*n* = 6). The density of NPY-Y1 receptor puncta/µm^3^ was determined using immunohistochemistry and image analysis of 3D rendered images from z-stacks using IMARIS software. (**d**) Two-way ANOVA repeated-measures comparison of NPY-Y1 receptor density between cortical layers on upper motor neurons from controls and ALS case motor cortex. Values in bars represent the average values of individual cases and controls. Bars represent the group mean ± SEM. ** *p* < 0.01. Scale bar = 10 µm.

The activity and function of motor neurons rely upon appropriate innervation of subcellular domains, thereby differentially regulating input, integration and output. This includes the dense perisomatic innervation received by upper motor neurons from within layer 5 of the motor cortex, which strongly influences cell output [47], and the axo-dendritic innervation received from layer 2/3 cells onto upper motor neuron apical dendrites, which influences temporal summation [48,49,50]. In the post-mortem ALS patient motor cortex, upper motor neurons undergo marked cellular and dendritic degeneration, which includes reduced numbers of excitatory post-synaptic connections on apical processes [51,52]. There is also evidence from animal studies that appropriate inhibitory inputs to upper motor neurons are lost in layer 5 of the motor cortex [19,24]. However, in the human ALS motor cortex, the density of inhibitory connections on upper motor neurons, and more specifically NPY receptors, remains to be investigated. We therefore began our investigation by characterizing the expression of NPY-Y1 receptors on the soma and apical dendrites of SMI32-positive upper motor neurons in the post-mortem motor cortex of ALS cases and non-ALS age-matched controls (Figure 2).

In the cohort examined, there was no significant differences between groups in age, sex or post-mortem interval (PMI) (*p >* 0.05; Table 3), although some variation in PMI is noted. Immunohistochemistry revealed that NPY-Y1 receptors localized to the soma and neurites of SMI32-positive upper motor neurons (Figure 2). Using one-way ANOVA, we found that there was a 20% increase in NPY-Y1 receptor density on upper motor neuron soma in ALS cases compared to controls. However, this was not quite statistically significant (F(1,13) = 3.680, *p* = 0.07) (Figure 2a). We next assessed NPY-Y1 receptor density on upper motor neuron apical dendrites in layer 4/5 (Figure 2b) and layer 2/3 (Figure 2c). We found that there was no significant difference in the NPY-Y1 receptor density between cases and controls in either layer 2/3 (F(1,6) = 2.143, *p* = 0.194) or layer 4/5 (F(1,6) = 0.013, *p* = 0.915) (Table 3). To determine differences in NPY-Y1 receptor distribution between upper motor neuron compartments, we next performed a two-way repeated-measures ANOVA. We found a significant main effect of upper motor neuron compartment on NPY-Y1 receptor density (F(2,25) = 9.412, *p* = 0.0009) (Figure 2d).

Tukey’s multiple comparisons test identified a significant 90% increase in NPY-Y1 receptors on the layer 2/3 apical dendrites of upper motor neurons compared to the layer 5 somatic compartment in controls (*p =* 0.0021; Figure 2d). This distinction between soma and layer 2/3 apical dendrite NPY-Y1 receptor density was not present in ALS cases (*p* > 0.05). Overall, these results suggest an increase in the density of somatic NPY-Y1 receptors on upper motor neurons in ALS cases.

### 3.2. NPY-Y1 Receptors Are Increased on SOD1^G93A^ Excitatory Cortical Neurons In Vitro

The hyperexcitability and vulnerability of cortical neurons have been documented in cortical neuron culture and pre-symptomatically in rodent models of ALS [12,13,53,54,55], suggesting that cortical neurons develop pathological alterations from very early stages in the disease. As such, we next assessed NPY-Y1 receptor expression in primary neocortical cultures derived from the *SOD1^G93A^* mouse model of familial ALS, to determine whether NPY-Y1 receptor alteration is an early feature of cortical neuron vulnerability.

**Figure 3:**
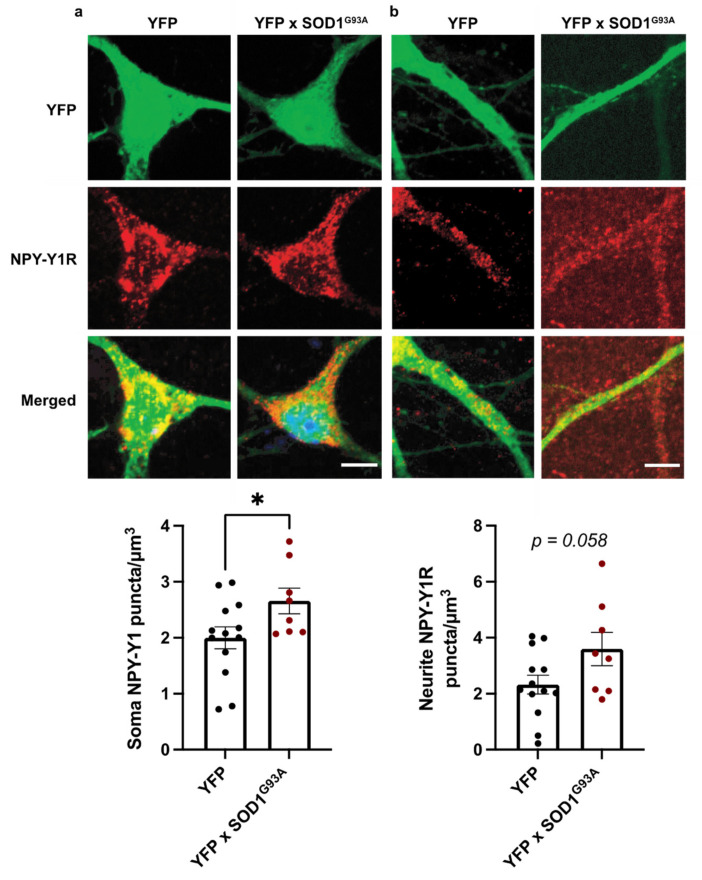
Immunocytochemical labelling of NPY-Y1 receptors (red) on the soma (**a**) and primary neurites (**b**) of cortical *YFP*-positive excitatory neurons (green) derived from *YFP* (*n* = 13) and *YFP::SOD1^G93A^* (*n* = 8) E15.5 mouse embryos. NPY-Y1 receptor puncta/µm^3^ was determined using immunocytochemistry and image analysis of 3D rendered images from z-stacks using Imaris software. Values depict the average NPY-Y1 receptor density of individual embryos. Bars represent the group mean ± SEM * *p* < 0.05. Scale bar = 5 µm.

Immunohistochemistry revealed that NPY-Y1 receptors localized to the soma and neurites of YFP-positive excitatory neurons (Figure 3). Quantification of receptor expression demonstrated a significant increase in NPY-Y1 receptors on the *YFP::SOD1^G93A^* cortical neuron soma by 54% compared to *YFP* neurons (F(1, 19) = 4.546, *p* = 0.046) (Figure 3a). In addition, quantification of receptor puncta along primary neurites indicated a 32% increase on *YFP::SOD1^G93A^* cortical neurons compared to *YFP* neurons; however, this was not quite statistically significant (F(1, 19) = 4.068, *p* = 0.058) (Figure 3b). Together, these results indicate that NPY-Y1 receptors are involved in post-synaptic dysfunction of cortical neurons, as an early feature of disease in the *SOD1^G93A^* model of ALS.

### 3.3. NPY-Y1 Receptor Density Is Modified on Distal Apical Dendrites of Upper Motor Neurons in a SOD1^G93A^ Mouse Model

**Figure 4:**
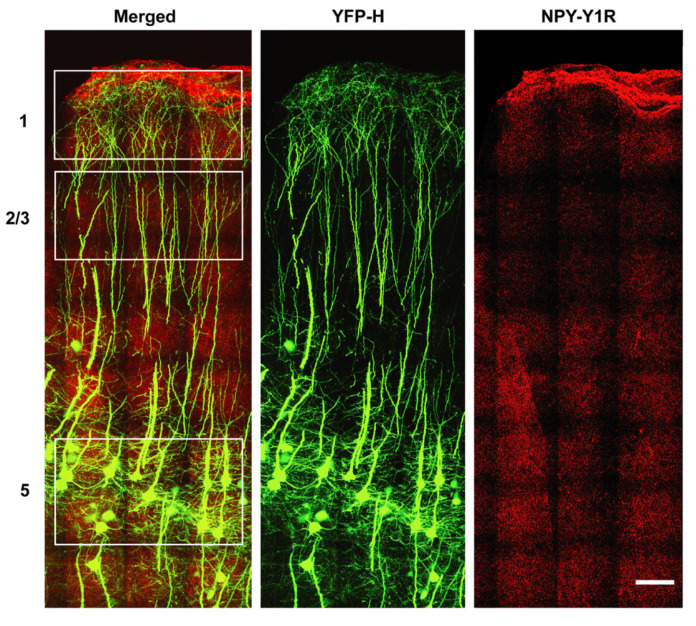
Representative immunohistochemistry of NPY-Y1 receptors (red) and YFP-H-positive upper motor neurons (green) obtained from a *YFP-H* mouse at 20 weeks of age. White boxes indicate regions selected for quantitative analysis of NPY-Y1 receptor expression on YFP-H upper motor neurons. Nissl stain was also utilized for lamina localization. Scale bar = 200 µm.

**Figure 5:**
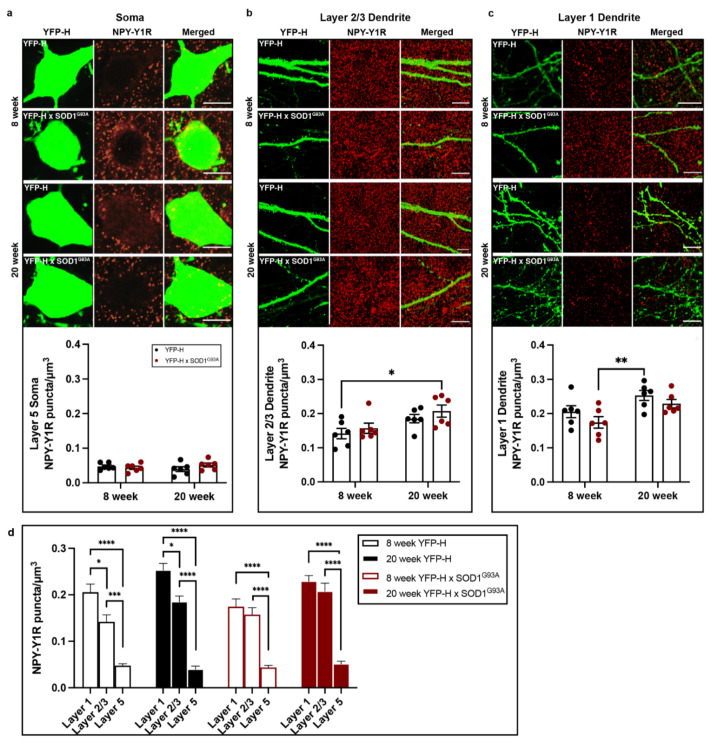
Immunohistochemical investigation of NPY-Y1 receptor density on layer 5 upper motor neurons throughout disease progression in *YFP-H::SOD1^G93A^* (*n* = 6 per age group) and *YFP-H* mice (*n* = 6 per age group). (**a**–**c**) Immunohistochemical comparisons of NPY-Y1 receptors (red) localised to the cell soma (**a**) and distal apical dendrites in layers 2/3 (**b**) and 1 (**c**) of YFP-H- (green) positive upper motor neurons between *YFP-H* and *YFP-H::SOD1^G93A^* mice at 8 and 20 weeks of age. (**d**) Three-way ANOVA comparisons of NPY-Y1 receptor density between cortical layers of *YFP-H* and *YFP-H::*SOD1^G93A^ mice at 8 and 20 weeks of age. Presented values represent the mean density for individual animals across a minimum of two brain slices. Bars represent the mean ± SEM. * *p* < 0.05, ** *p* < 0.01, *** *p* < 0.001, **** *p < 0.0001*. Scale bar = 10 µm.

To understand whether increased NPY-Y1 receptor density persists throughout disease, of fluctuates with key stages of disease, we investigated layer 5 upper motor neurons in the motor cortex of the *SOD1^G93A^* mouse at an early-symptomatic (8 week) and late-symptomatic (20 week) time point. In the *SOD1^G93A^* motor cortex, we previously identified distinct changes in NPY-positive cells at these early and late time points [27]. For this study YFP-H expression in transgenic *Thy1-YFP-H* mice was used to visualize upper motor neurons and their processes in the rodent motor cortex (Figure 4). Initial assessment of somatic NPY-Y1 receptor labeling on YFP-H-positive upper motor neuron soma showed no significant difference between *YFP-H* controls and *YFP-H::SOD1^G93A^* mice at either 8 or 20 week time points (Figure 5a; *p >* 0.05).

Similarly, analysis of NPY-Y1 receptor density on upper motor neuron apical processes in layer 2/3 showed no differences between *YFP-H::SOD1^G93A^* mice and *YFP-H* mice at either time point (Figure 5b). However, there was a main effect of age on NPY-Y1 receptor density in layer 2/3 (F(1, 20) = 9.633, *p =* 0.0056). Tukey’s multiple comparisons test indicated that there was no statistically significant difference between *YFP-H::SOD1^G93A^* and *YFP-H* animals at either time points, with the exception of a statistically significant increase between 8 week *YFP-H* animals and 20 week *YFP-H::SOD1^G93A^* mice (Figure 5b, *p =* 0.0278).

We next analyzed NPY-Y1 receptor expression on YFP-H-positive upper motor neurons apical processes that extend into layer 1. NPY-expressing neurons are predominantly situated in superficial cortical layers 1 and 2, where their horizontal-orientated projections have been shown to release NPY onto distal apical dendrites of pyramidal neurons [56,57]. It had not previously been possible to assess receptor density on layer 1 processes in human post-mortem tissue, since layer 1 upper motor neuron apical processes could not be distinguished from layer 2/3 pyramidal neuron apical processes, including in normal controls, with SMI32 immunohistochemistry. Using two-way ANOVA, we demonstrate a main effect of age on post-synaptic NPY-Y1 receptor density on layer 1 dendrites (F(1, 20) = 11.05, *p* = 0.003) (Figure 5c). Tukey’s multiple comparisons test identified this effect was driven by a 42% increase in NPY-Y1 receptors on layer 1 apical processes in 20 week *YFP-H* mice compared to 8 week *YFP-H::SOD1^G93A^* animals (Figure 5c, *p* = 0.0086). Interestingly, this increase in receptors with age was also observed in 20 week *YFP-H::SOD1^G93A^* animals compared to 8 week *YFP-H::SOD1^G93A^* animals, although it did not quite reach statistical significance (*p* = 0.088).

As the density of receptors appeared to differ between the distinct domains of the upper motor neuron soma and processes, we next performed a three-way ANOVA analysis to determine the effect of genotype, age and upper motor neuron compartment on receptor density. We confirm significant differences in NPY-Y1 receptor density on upper motor neuron domains in the different cortical layers (F(2, 60) = 189.5, *p* = 0.0001) (Figure 5d). Notably, there was significantly fewer NPY-Y1 receptors in contact with the soma of upper motor neurons (at least 70% less) compared to the apical dendrites in layer 2/3 and 1 (*p* < 0.05). This occurred at all time points in both genotypes, suggesting a physiological difference between the extent of NPY signaling received by upper motor neurons on the apical dendrites compared to the cell soma.

Interestingly, post hoc tests identified a significant difference in the density of NPY-Y1 receptors on upper motor neuron apical processes in cortical layer 1 compared to layer 2/3 of *YFP-H* animals at both 8 and 20 week time points (Figure 5d, *p* < 0.05). However, the distinction between these dendritic compartments was lost in the *SOD1^G93A^* model at both time points (*p* > 0.05). Indeed, three-way ANOVA analysis revealed a statistically significant interaction between cortical layer and genotype (F(2, 60) = 3.414, *p* = 0.0394) (see Table A1). Suggesting that NPY-Y1 receptor density is changed on the distal apical dendrites of upper motor neurons in the *SOD1^G93A^* motor cortex from 8 weeks of age; a pattern of receptor distribution that remains by end stages of disease at 20 weeks. Three-way ANOVA analyses also highlighted an age dependent effect of cortical layer on NPY-Y1 receptor density (F(1, 60) = 19.38, *p* = 0.0001), as well as interaction of cortical layer and age on receptor density (F(2, 60) = 4.914, *p* = 0.0106). Collectively, these results indicate that the normal distribution of NPY-Y1 receptors is distinct on upper motor neuron domains, with the density of receptors found in layer 1 > layer 2/3 and layer 5 soma. However, this distinctive pattern of receptor distribution appears to be lost on upper motor neurons in the *SOD1^G93A^* motor cortex, particularly on the apical dendrites of upper motor neurons.

## 4. Discussion

This study provides evidence for age- and disease-associated changes in the expression of NPY-Y1 receptors on upper motor neurons in post-mortem ALS tissue and in the *SOD1^G93A^* mouse model of ALS. Specifically, we demonstrate increased NPY-Y1 receptor density on the soma of layer 5 human ALS upper motor neurons and also in vitro in *SOD1^G93A^* mouse cortical neurons. Interestingly, evidence from the *SOD1^G93A^* motor cortex indicates the distribution of NPY-Y1 receptor density is altered on the apical processes of upper motor neurons at both an early-symptomatic and late-symptomatic stage of disease. Collectively, these data suggest that while NPY-Y1 receptors appear to be modified on disease-affected upper motor neurons, their presence on key subcellular domains of the cell throughout the disease, indicates they may be targeted to promote NPY-mediated neuroprotective actions in the ALS motor cortex.

Investigations in the post-mortem motor cortex of ALS cases and controls confirmed the presence of post-synaptic NPY-Y1 receptors on the soma and apical dendrites of layer 5 upper motor neurons. These cellular compartments have important roles in regulating the input, integration and output of upper motor neuron signaling essential for the initiation of movement by downstream motor pathways [48,49,50]. Neuroprotective effects of NPY have included the improvement of motor deficits and survival in a model of neurodegenerative disease [58,59,60]. In line with previous work from the field [27,28], we find evidence for NPY system involvement in the ALS pathogenesis. Specifically, our evidence suggests an increase in NPY-Y1 receptors on the soma of upper motor neurons in the post-mortem motor cortex of ALS cases. While this work indicates modification of this receptor in the disease, the sustained presence of NPY-Y1 receptors on the key cellular compartments of layer 5 upper motor neurons highlights a potential target for NPY system modulation. At present, several selective NPY receptor agonists and antagonists have been developed and are widely used in research [61], while nasal delivery of the NPY peptide has been trialed for people with post-traumatic stress disorder [62]. Neuroprotection conferred by the NPY system has been demonstrated through modulation of neurotrophic pathways, neuroinflammation, pathogenic excitability, endoplasmic reticulum stress and mis-regulation of autophagy, mechanisms which have been implicated in the ALS pathogenesis (see review [26]).

In support of our findings on ALS upper motor neurons in human post-mortem motor cortex, our in vitro investigations show that NPY-Y1 receptors are modified on the soma of cortical glutamatergic neurons in the *SOD1^G93A^* ALS model. However, we also find evidence for increased NPY-Y1 receptors on the neurites of cortical neurons at this relatively early time point in disease progression in the *SOD1^G93A^* model. Previous in vitro investigations have demonstrated a number of early intrinsic modifications that affect the normal function of synapses and ion channels in not only the *SOD1* model but also in other familial ALS models and patient-derived induced pluripotent stem cells [9,53,63,64,65]. Our data suggest that modification of the post-synaptic NPY-Y1 receptors is also involved in these early changes that demonstrate, and possibly influence, cortical neuron vulnerability in the disease. Given that changes to NPY-Y1 receptors was not present on the apical dendrites in post-mortem ALS motor cortex, this may also indicate involvement of extrinsic factors in late-stage pathology that influence NPY-Y1 receptor distribution on the upper motor neuron. It is known that the apical dendrites of the upper motor neuron display significant degeneration in the post-mortem ALS motor cortex [52], while subtle dysfunction is present on cortical neurons in vitro [9,53,63].

In this study, we found clear evidence of increased post-synaptic NPY-Y1 receptor inputs onto the upper apical processes of layer 5 upper motor neurons relative to the somatic compartment in the motor cortex of humans and rodents. This is in line with previous literature that shows that NPY interneurons are predominantly distributed between superficial cortical layers 1–3 and release NPY into cortical layer 1 via horizontal processes [56,66]. Functionally, this may reflect the degree of synaptic innervation required for subcellular compartments to appropriately influence the output of upper motor neuron signaling [47]. Furthermore, we observed an age associated increase to the density of NPY-Y1 receptors in the rodent motor cortex between 8 and 20 weeks of age which could suggest that this receptor has a key role in adaptive plasticity mechanisms of the motor cortex.

Interestingly, we found that the distinct subcellular compartment distribution of NPY-Y1 receptors was lost in the *SOD1^G93A^* motor cortex. Specifically, there was a loss of distinction between receptor densities of upper motor neuron apical dendrites between cortical layers 1 and 2/3. However, there was no overt differences in somatic NPY-Y1 receptor densities, as was previously demonstrated in the human post-mortem motor cortex. While these data may suggest subtle differences between human pathology and the transgenic model utilized in this study, it may also be explained by wider involvement of the NPY system in the motor cortex.

Critically, we previously showed that there was an early decrease in the number of NPY interneurons in the motor cortex of the *SOD1^G93A^* mouse at 8 weeks of age [27]. This decrease was specific to the upper cortical layers of the motor cortex (layer 1–4) and was not demonstrated in the lower cortical layers (layer 5–6), which may explain the modification of apical NPY-Y1 receptors and the lack of somatic changes observed in this model. While future studies should assess changes to NPY interneurons in the human motor cortex, it is also important to note that changes to the distribution of receptors supports a broader vulnerability of this motor cortex region. Apical dendrites of upper motor neurons have previously been shown to undergo marked degeneration specifically within layer 2/3 of the SOD1^G93A^ mouse motor cortex from P60 (~8 weeks of age) [67]. Collectively, this may suggest a relationship between apical dendrite vulnerability, post-synaptic NPY-Y1 receptor expression and NPY system function in the disease pathogenesis, which should be explored pre-symptomatically in future studies and extended to include basal dendrites that preferentially receive excitatory inputs from other brain structures, such as the secondary motor cortex [68]. Nonetheless, given the number of studies suggesting a neuroprotective role for NPY in neurodegenerative diseases and the availability of receptor specific modulators [33,69], future studies should consider the targeted manipulation of these post-synaptic receptors as a novel avenue to influence upper motor neuron function in the disease.

## 5. Conclusions

Overall, this work demonstrates the presence of NPY-Y1 receptors on the upper motor neurons in a rodent model of ALS and in the post-mortem motor cortex. We find evidence to support NPY system involvement in the disease pathogenesis, with changes to the distribution of NPY-Y1 receptor density on the soma and apical dendrites in both the SOD1^G93A^ and ALS motor cortex. While some discrepancies are observed between human and rodent models, a clear pattern of receptor distribution is found on the upper motor neuron, which given the role of NPY in conferring neuroprotection in neurodegenerative diseases, makes this system and this receptor worthy of future studies that aim to explore the potential to modulate motor neuron function to alleviate motor symptoms in ALS.

## Figures and Tables

**Table 1:** ALS case and control demographics.

Diagnosis	Sex	Age at Death, Year	Post-Mortem Interval, Hour
ALS 1	M	78.1	13.5
ALS 2	F	69.3	45
ALS 3	F	67	25
ALS 4	M	62	29
ALS 5	M	63.9	14
ALS 6	M	65.2	13.5
ALS 7	F	74.4	7
ALS 8	M	62	12.5
ALS 9	M	55	8
Control 1	F	56	28
Control 2	M	48	17
Control 3	M	73	38.5
Control 4	F	67.3	24
Control 5	M	64.1	24
Control 6	M	63.9	68

Abbreviations: F = Female, M = Male.

**Table 2:** Immunohistochemistry primary and secondary antibody table.

Primary Antibody	Company	Species	Category Number (RRID)	Dilution Factor		
				Human	Mouse Tissue	Mouse In Vitro
Anti-NPYY1R	Genetex	Rabbit	GTX54639 (AB_2887869)	1:150	1:200	1:1000
Anti- neurofilament-H Nonphosphorylated SMI32	Biolegend	Mouse	801701 (AB_2564642)	1:500		
Secondary Antibody	Company	Species	RRID	Dilution Factor		
AlexaFluor 488	Molecular Probes	Mouse	AB_2576208	1:1000		
AlexaFluor 546	Molecular Probes	Rabbit	AB_2534093	1:1000		
AlexaFluor 594	Molecular Probes	Rabbit	AB_2650602	1:1000		

**Table 3:** Comparison of ALS case and age-matched control characteristics.

Characteristic	Control (*n* = 6)	ALS (*n* = 9)	*p* Value
Age at death, years	62.05 (8.8)	66.32 (6.94)	0.313
Male	4 (66%)	6 (66%)	0.706 ^1^
PMI, hours	33.25 (18.4)	18.66 (12.31)	0.087
Y1R density (µm)^3^ × L5 Soma	0.24 (0.04)	0.29 (0.04)	0.077
Y1R density (µm)^3^ × *L4/5 Dendrite	0.31 (0.08)	0.32 (0.13)	0.915
Y1R density (µm)^3^ × *L2/3 Dendrite	0.44 (0.07)	0.38 (0.05)	0.194

Continuous variables are expressed as the mean ± (SD) and categorical variables are expressed as *n* (%). *p* values are based on ^1^ Fisher’s exact test (sex), other ANOVA (age at death, PMI and Y1R density). Abbreviations: PMI = post-mortem interval; Y1R = neuropeptide Y1 receptor * dendrite analyses utilized *n* = 3 controls and *n* = 5 ALS cases.

## Data Availability

The data presented in this study are available upon reasonable request from the corresponding author.

## Appendix A

**Table A1:** Three-way ANOVA of factors influencing NPY-Y1 receptor density on upper motor neurons within the mouse motor cortex.

Factor	F	*p* Value
Cortical layer	F(2, 60) = 189.5	0.0001
Genotype	F(1, 60) = 0.0459	0.8310
Age	F(1, 60) = 19.38	0.0001
Cortical layer × Genotype	F(2, 60) = 3.414	0.0394
Cortical layer × Age	F(2, 60) = 4.914	0.0106
Genotype × Age	F(1, 60) = 0.4569	0.5017
Cortical layer × Genotype × Age	F(2, 60) = 0.03959	0.9612
